# Metabolomic profiling and 16 S rRNA metabarcoding of endophytes of two *Aloe* species revealed diverse metabolites

**DOI:** 10.1186/s13568-024-01784-3

**Published:** 2024-11-08

**Authors:** Cynthia Marokane-Radebe, Adekunle Raimi, Stephen Amoo, Rasheed Adeleke

**Affiliations:** 1 Unit of Environmental Sciences and Management, North-West University, Potchefstroom, 2520 South Africa; 2 Agricultural Research Council - Vegetables, Industrial and Medicinal Plants, Roodeplaat, Pretoria, 0001 South Africa

**Keywords:** Secondary metabolites, Endophytic bacteria, Endangered, Biotechnology, Medicinal plant

## Abstract

*Aloe* species are often used interchangeably for medicinal and cosmeceutical applications, presenting a challenge to the biological efficacy consistency of some herbal preparations. Sustainable production of high-quality commonly used medicinal plants remains a limitation for commercialisation. Thus, this study investigated the potential for plant substitution by examining bacterial endophytes capable of producing similar host plant secondary metabolites. The metabolite profiles and endophytic bacterial communities of endangered *Aloe lettyae* were compared with those of *Aloe longibracteata* using nuclear magnetic resonance spectroscopy and 16 S rRNA gene sequencing. Only 15 metabolites were significantly different between *A. lettyae* and *A. longibracteata* based on metabolite concentrations. However, both plants’ functionality and potential application remain comparable. Phytohormones, including indole-3-acetate and 5-hydroxyindole-3-acetate, were more concentrated in *A. lettyae* than *A. longibracteata*. Metabolites such as tyrosine, allantoin, and myo-inositol, with human health benefits, were annotated in both species. *Aloe lettyae* harboured a phylogenetically diverse bacteria community compared to *A. longibracteata*, with a higher richness of bacterial species, indicating a likelihood of diverse metabolic capabilities among the bacteria. Dominant endophytes, including *Bacillus*,* Comamonas*, and *Pseudomonas*, possess enzymes contributing to various metabolic pathways. The enzymes have the potential to impact the synthesis, or breakdown of plant metabolites, consequently influencing the overall metabolic composition of the host plant. Therefore, this study supports the interchangeability of *A. lettyae* and *A. longibracteata* due to their ability to produce similar metabolites, and although the *Aloe* species exhibit phylogenetically diverse endophytic communities, the feasibility of utilizing their endophytes as producers of secondary metabolites remains viable.

**Graphical abstract:**

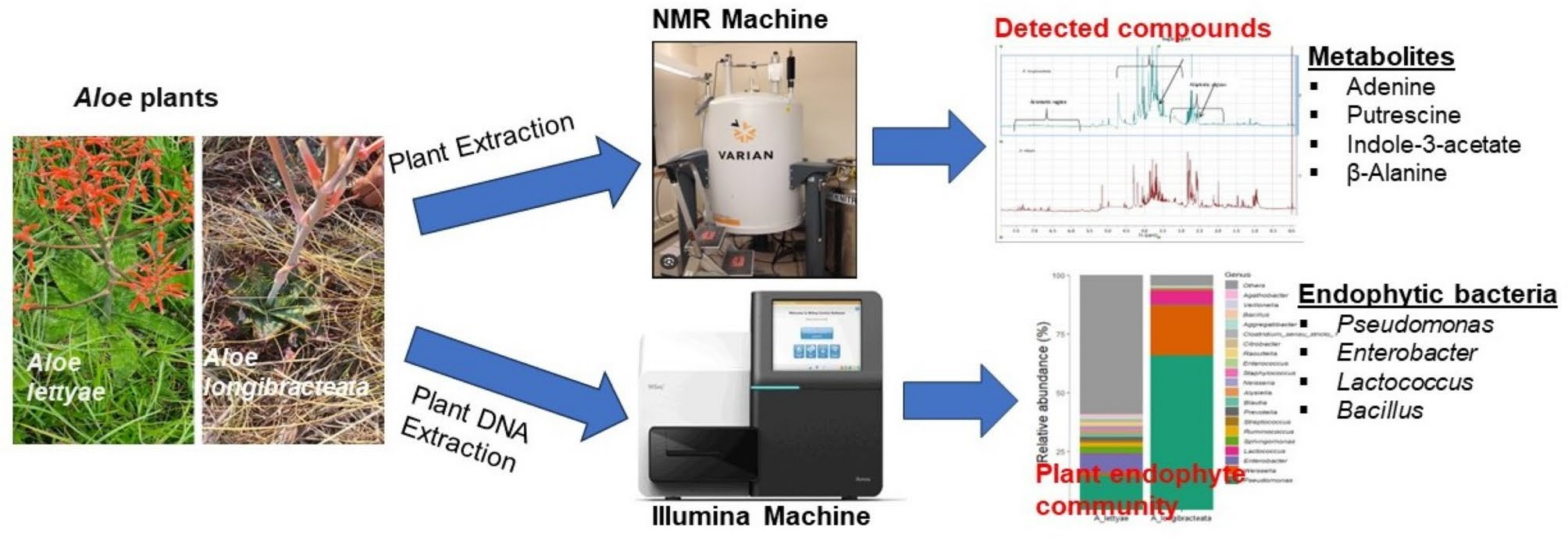

**Supplementary Information:**

The online version contains supplementary material available at 10.1186/s13568-024-01784-3.

## Key points

- *Aloe* plants harbour bacterial endophytes with biotechnological importance.
- *A. lettyae* can be exchanged for *A. longibracteata* due to their similar metabolites.
- Bacterial endophytes produced phytohormones with plant growth-promoting potential.

**Supplementary Information:**

The online version contains supplementary material available at 10.1186/s13568-024-01784-3.

## Introduction

In medicine, biotechnology and pharmacology, there is a controversy about the nature and type of bioprospecting approach that can be adopted for medicinal plants to guide drug discovery (Saslis-Lagoudakis et al. 2012). Efficacy and sustainable production of traditionally used medicinal plants are two of the most important concepts driving research interests in the bioprospecting of medicinal plants. For instance, perceived efficacy is strongly influenced by cultural biases linked to ethnobotanical data, especially when similar plants are used for the same purpose in different regions (Saslis-Lagoudakis et al. 2011). However, over-reliance on ethnobotanical data may not present complete information about the potential benefits and bioactivity of certain medicinal plants (Saslis-Lagoudakis et al. 2011). This means ethnobotanical data comparisons of medicinal plants could only be used as a guide in bioprospecting if accompanied by relevant data, especially the nature and quantity of metabolite production by the medicinal plants. Interestingly, recent developments in drug discovery have expanded the focus beyond plant metabolites and there is an increasing interest in the plant endosphere that harbours the endophytes. Endophytes are known to have the capability to produce similar categories of metabolites as their host plants (Kusari et al. 2013; Ludwig-Müller 2015). In addition, endophytes can produce unique and novel bioactive compounds of biotechnological importance (Ambrose et al. 2013; Gouda et al. 2016; Uzma et al. 2019). A powerful but underutilised approach to address challenges associated with the sustainable production of high-quality bioactive medicinal plants could be through an unbiased comparison of plants used for the same medicinal purposes. This could be achieved by focusing on the metabolites from closely related plant species and their native microbiome, especially the endophytes.

The genus *Aloe* comprises over 500 species of succulent plants renowned for their medicinal properties and widespread use in traditional medicine (Grace et al. 2008). Among these, *Aloe lettyae* (*A. lettyae*) and *Aloe longibracteata (A. longibracteata)* are closely related species native to South Africa (Smith and Klopper 2022). They belong to the group of spotted aloes, a group with species not easily distinguishable, and taxonomically difficult to identify at the vegetative phase (Van Wyk and Smith 2014) However, *A. lettyae* is classified as an endangered species due to habitat loss, abiotic disturbances such as fire and the effect of the abundance of interacting species (Kremer-Köhne et al. 2020). Therefore, sustainable production of endangered plant species such as *A. lettyae* is necessary to protect the plants from extinction. In addition, associated microbiomes, especially the microbial endophytes, known to possess the ability to influence the production of secondary metabolites and yield in aloe plants should be investigated.

Endophytic microbes could be applied to improve plant biomass and/or the quantities of metabolites produced by host plants. In addition, plant growth-promoting endophytes could help protect the plants against some negative effects of abiotic and biotic factors (Raimi et al. 2017), thereby improving their resilience and growth under suboptimal growing conditions. Furthermore, the isolation and characterisation of endophytes associated with medicinal plants may provide insights into their capabilities to synthesise secondary metabolites similar to that of their host plants (Pimentel et al. 2011).

Plant metabolites play crucial roles in various physiological processes, defence mechanisms and ecological interactions (Pichersky and Gang 2000). Metabolomic approaches have been employed to characterise the chemical diversity of *Aloe* species, revealing the presence of diverse metabolites such as anthraquinones, chromones, and polysaccharides (Cock 2015; Sahu et al. 2013). In addition to their intrinsic metabolic capabilities, aloe plants harbour diverse microbial communities, including endophytic bacteria that inhabit their internal tissues without causing apparent harm (Hardoim et al. 2015). These endophytic bacteria contribute to plant growth and development, through nutrient acquisition and phytohormone production (Raimi and Adeleke 2023; Santoyo et al. 2016). Endophytes have been shown to influence plant secondary metabolite synthesis, leading to the production of unique and analogous metabolites with potential bioactivities (Kumari et al. 2023). Thus, insight into the endophytic bacterial communities of *Aloe* species may improve our knowledge of the correlations between the metabolites of the host plant and its associated endophytic bacteria.

Despite studies about metabolite production and associated endophytic bacterial communities of aloe plants, results have had limited impacts on potential biotechnological applications (Silva et al. 2020; Swati et al. 2022). If *A. lettyae* and *A. longibracteata* harbour bacterial endophytes capable of synthesising metabolites analogous to those of the host plant, there might be less need to rely on the harvesting of plant material for metabolite sourcing, particularly in the case of the endangered *A. lettyae*. Thus, this study hypothesized that plants from the same lineages utilised for the same purpose produce similar sets of metabolites and harbours similar types of bacterial endophytes. Furthermore, the study hypothesised that such endophytes have the potential to produce similar metabolites as that of their host plants. If these hypotheses are correct, then such plant groups should be strong candidates for bioprospecting. To our knowledge, this is the first study that aims to gain insight into the correlation between the differential metabolites and endophytic bacterial communities of *A. lettyae* and *A. longibracteata.* The results may provide baseline information for the unique utilisation of specific aloe species and improve microbial technology applications, especially in the production of bioactive compounds using endophytic microbes.

## Materials and methods

### Plant sampling area

**Fig. 1:**
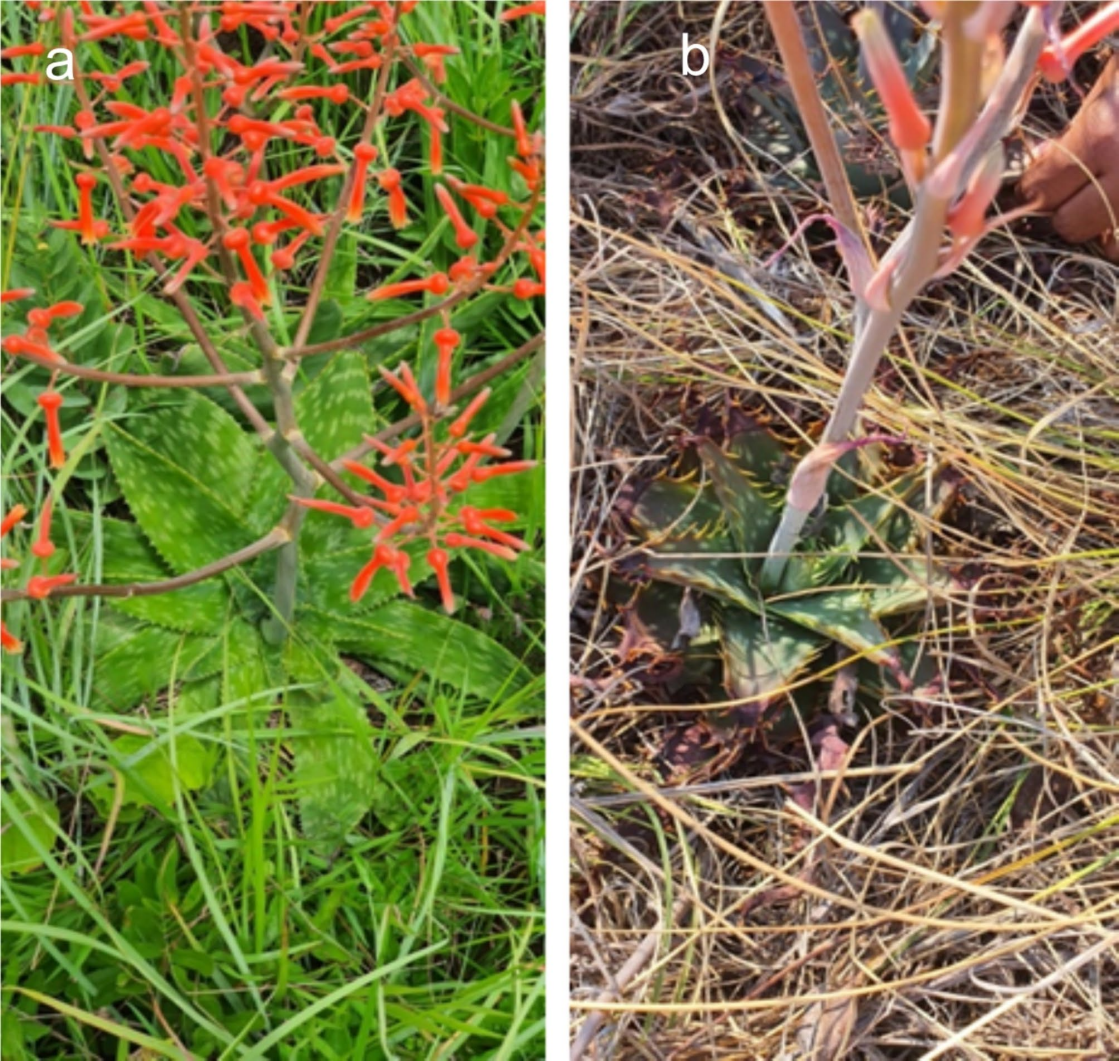
Sampled *Aloe* species. **a** *A. lettyae* and **b** *A. longibracteata* collected from Haenersburg and Makapanstad villages in Limpopo province, South Africa

*Aloe lettyae* and *A. longibracteata* (family *Asphodelaceae)* were collected from Haenersburg (23° 55’ 59.99” S 29° 56’ 59.99” E) and Makapanstad (25° 14’ 35” S, 28° 7’ 18” E) respectively, in Limpopo Province, South Africa. Six individual plants from each species were collected per site (approximately 200 m apart). The plant samples were carefully uprooted and placed in sterile polyethylene bags and brought to the laboratory in a portable cooler maintained at 4ºC using ice packs. The formal identification of the plant specimens (Fig. 1) was carried out at the herbarium of the Botany Department at North-West University, where the plant herbarium specimens were deposited.

### Plant extraction and NMR analysis

Using the method of Kim et al. (2010) with modifications, the freeze-dried powdered leaf samples (50 mg) of *A. lettyae* and *A. longibracteata* (Fig. 1) were extracted with 750 µl potassium dihydrogen phosphate (KH_2_PO_4_) and 750 µl methanol-d_4_ (CH_3_OH-d_4_) buffer in deuterium water (D_2_O) (pH 6.0) that contained 0.01% (w/w) trimethylsilanepropionic acid (TSP). The mixture was vortexed for 1 min, ultrasonicated for 20 min, and then centrifuged for 20 min at 10,000 rpm. After filtering through a 0.22 μm syringe, 500 µl of the filtrate was transferred to 5 mm Norell standard NMR tubes. A 600 MHz NMR spectrometer was used to acquire all the proton NMR spectra (Varian Inc, Palo Alto, CA, USA). Gradient shimming was used to improve the magnetic field homogeneity prior to all acquisitions. All spectra were Fourier-transformed, and phase and baseline were corrected manually.

### Multivariate data analysis

MestReNova software (10.0.1, Mestrelab Research, Spain) was used for data analysis while correction of phasing and baseline, normalisation and peak alignment were done manually on the 1 H NMR spectrum. The processed data was divided into 0.04 ppm bins, representing 0–10 ppm and converted to Excel comma-separated values (CSV) file format for pattern recognition multivariate data analysis. After transformed data were statistically analysed, the remaining imported data were Pareto scaled in the soft independent modelling of class analogy (SIMCA) software (Version 14.0, version 14.0, Sartorius Stedim Data Analytics AB, Umetrics, Umeå, Sweden). The Principal Component Analysis (PCA) and orthogonal projections to latent structure discriminant analysis (OPLS-DA) were employed to distinguish between similar and non-similar samples. The compounds were annotated using the Chenomx software (NMR suite, version 8.3, Edmonton, AB, Canada) and published NMR data.

### Endophytic bacterial community composition of *A. lettyae* and *A. longibracteataas* revealed by high-throughput sequencing of the 16 S rRNA gene

#### Library preparation

Genomic DNA was extracted from the two *Aloe* species leaves using the Qiagen DNeasy Plant kits (QIAGEN^®^, Hilden, Germany), according to the manufacturer’s instructions. The DNA quantification was done with a Nanodrop™-1000 (Nanodrop lnc., Wilmington, USA), and the integrity and size were checked with electrophoresis using 1% agarose gel. Using Illumina barcoded primers targeting V3/V4 region, 341 F (5′-CCTACGGGNGGCWGCAG-3′) and 805R (5′-GACTACHVGGGTATCTAATCC-3′), the 16 S rRNA gene was amplified in a polymerase chain reaction (Klindworth et al. 2013). Following the method already described by Van Wyk et al. (2017), the library was prepared and sequenced on Illumina MiSeq sequencer as paired-end (250 bp) at the Novogene Sequencing facility, Singapore.

#### Bioinformatics analysis

Following demultiplexing, reads obtained were checked for quality with fastqc v. 0.11.9 (Babraham Bioinformatics, UK). Subsequently, the removal of chimaeras, correction for errors and clustering of the sequences at 100% similarities to amplicon sequence variance (ASVs) were carried out in Quantitative Insight into Microbial Ecology (QIIME) 2 software (Caporaso et al. 2010) with the DADA2 plugin. Phylogenetic relationship, and taxonomic classification, alpha and beta diversity were analysed in QIIME2 and R version 4.3.2 (R Development Core Team 2018). After subsampling of the ASV table to an even depth, the alpha diversity, based on Observed ASVs, Chao1, Shannon, Simpson and Pielou evenness, was used to analyse the community diversity, richness, and uniformity. The rarefaction curve was used to analyse the sequencing depth adequacy. Endophytic bacterial community composition and complexity were computed and compared between the different species of aloes using beta diversity, based on weighted and unweighted unifrac distances with non-metric multidimensional scaling (NMDS) in R. A Venn diagram was drawn using the ggvenn package in R to show the presence and distribution of core and shared species between the *Aloe* species. Core species are the ‘stable’ part of the community exhibiting key functions that impact host physiological performance (Alibrandi et al. 2020).

#### Functional profiling of endophytic bacteria community

Phylogenetic investigation of communities by reconstruction of unobserved states 2 (PICRUSt2) pipeline (version 2.3.0) was employed to compute the functional metabolic profile of the endophytic bacterial communities (Douglas et al. 2020). The analysis was performed in QIIME2 (Caporaso et al. 2010) using the ASV sequences and abundance table generated against the SILVA rRNA (138 release) (Quast et al. 2013) database as the inputs. The output files included gene family and pathway abundances (Douglas et al. 2020). The impacts of plant species on the bacterial community functions were assessed using the NMDS. A subset of essential pathways contributing to the synthesis of key metabolic compounds of ecological importance were analysed.

### Statistical analyses

A statistically significant level of *P* < 0.05 was used in this study and data analyses were performed in R version 4.3.2 (R Development Core Team 2018). The Shapiro-Wilk test was employed to establish if the data was normal and non-normal data was transformed using the square root or log functions. The parametric and non-parametric tests were adopted for normally and non-normally distributed data, respectively. The correlations between bacterial community composition and unique metabolic pathways were assessed with the Mantel test. With the vegan and dendextend (v. 1.12.0) in R, the bacterial community structure was visualized in multivariate space with a non-metric multidimensional scaling (NMDS) and an unweighted pair-group with arithmetic mean (UPGMA) technique. Differences between groups were established with weighted and unweighted unifrac distances dissimilarity. The most differentially abundant community and pathways between sample groups were identified using Linear Discriminant Analysis (LDA) Effect size (LEfSe) (Mann-Whitney U test, *P* < 0.05, LDA score > 2.0). Using the web-based Microbiome Analyst tool (www.microbiomeanalyst.ca), statistically significant and differentially abundant communities were visualized.

## Results

### Analysis of the metabolites produced by*Aloe*species

**Fig. 2:**
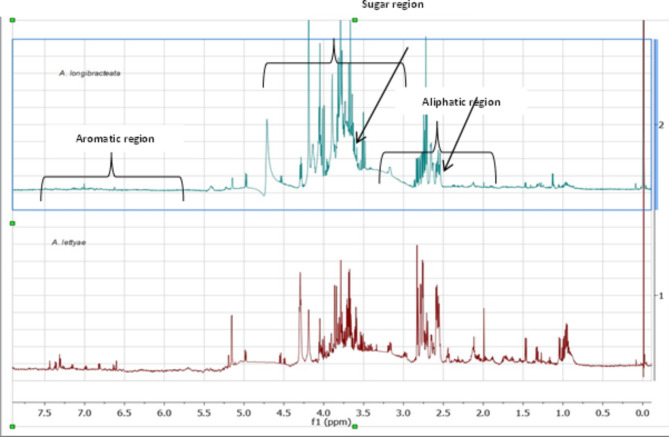
Stacked 1HNMR spectra of *Aloe longibracteata* and *Aloe lettyae.* The arrows show similar occurrences of metabolites between *A. longibracteata* (blue) and *A. lettyae* (red)

The 1 H NMR spectra of both *A. longibracteata* and *A. lettyae* revealed a complex array of signals, suggesting the presence of numerous metabolites ranging from amino, fatty, and organic acids in the aliphatic region, carbohydrates in the sugar region and phenolic metabolites in the aromatic region (Fig. 2). In the spectrum for *A. longibracteata*, there is a notable high intensity of signals across sugar and aliphatic regions (Fig. 2). In the sugar region (3-5.5ppm), the strong signal suggests a higher concentration of glycosylated metabolites and free sugars in *A. longibracteata* compared to *A. lettyae.* Similarly, the aliphatic region (0-3ppm) indicated a higher prevalence of metabolites such as fatty acids, organic acids, and other aliphatic metabolites in *A. longibracteata* as compared *to A. lettyae.* Despite the differences in the intensities of the metabolites in the sugar and aliphatic region of *A. longibracteata* and *A. lettyae*, these two *Aloe* species show similar signals in the aromatic region (6–8 ppm), indicating the presence of aromatic compounds like phenolics or aromatic amino acids in both species. Overall, the visual inspection of *A. longibracteata* and *A. lettyae* spectra indicates that *A. longibracteata* has a more diverse and abundant metabolite profile, particularly in terms of sugars and aliphatic metabolites compared to *A. lettyae*. Using the NMR profiles and plotting the PCA scores, the relative differences between *A. lettyae* and *A. longibracteata* were visualized.

**Fig. 3:**
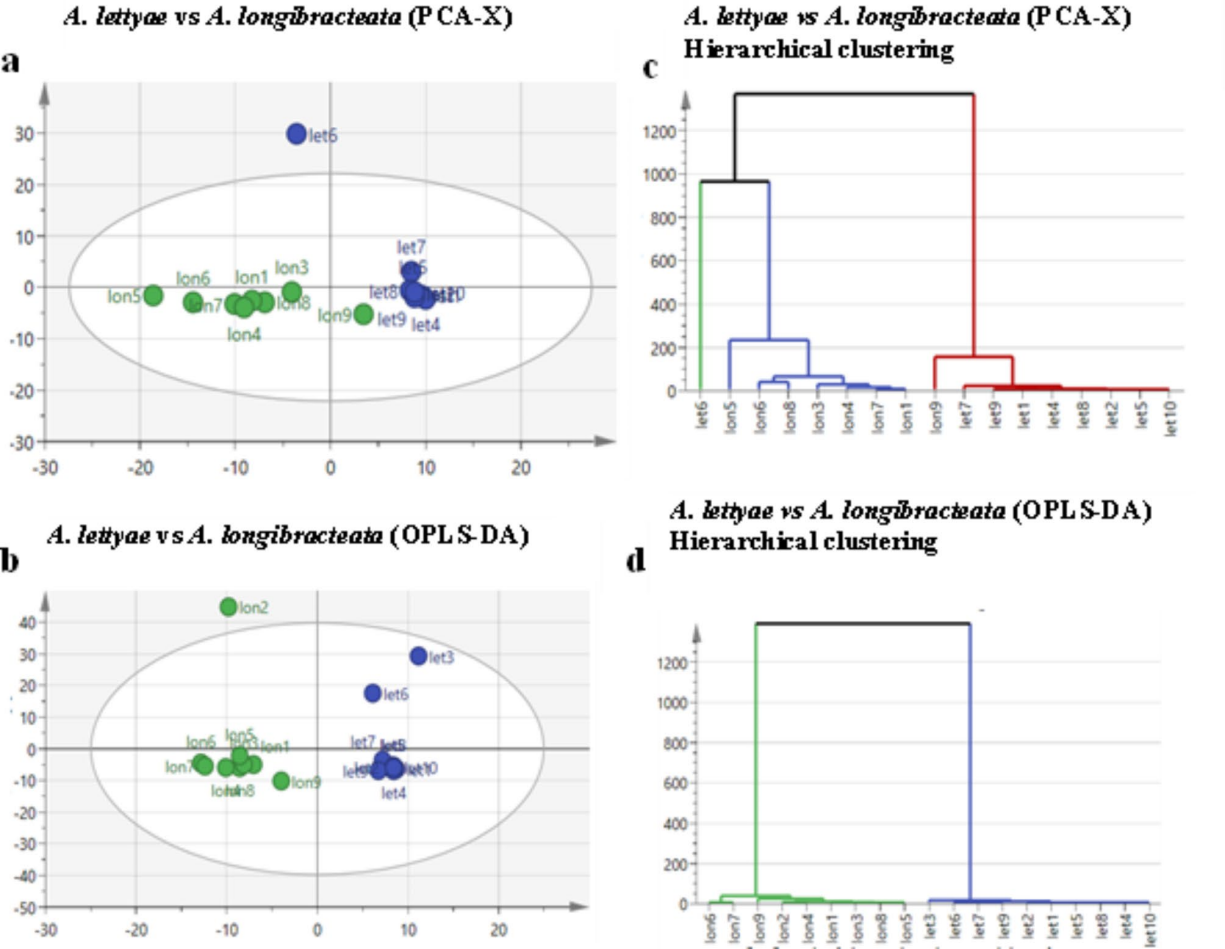
Unsupervised recognition analysis using **a** PCA and **b** OPLS-DA scores of *A. lettyae* and *A. longibracteata* aqueous methanol extract with HCA dendrograms derived from the **c** PCA and **d** OPLS-DA showing metabolic relativity of the samples, with green and blue circles representing *A. longibracteata* and *A. lettyae* samples, respectively

An overview of the sample dimensions was established by the unsupervised pattern recognition analysis using PCA (Fig. 3a). The data set obtained was subjected to the OPLS-DA, a supervised recognition analysis that enables the algorithm to reveal discrimination between the groups, (Fig. 3b). The 95% variance, coefficient R2X of 0.92 and Q2 of 0.72 were used to validate the goodness and predictability of the model. A distinct separation between samples was observed for the PCA scores plot (Fig. 3a) and the OPLS-DA score plot (Fig. 3b). Furthermore, the HCA dendrograms (Fig. 3c and d) grouped samples with similar features into three clusters, which suggests the distinction amongst the samples and reveals underlying patterns of the data.

**Fig. 4:**
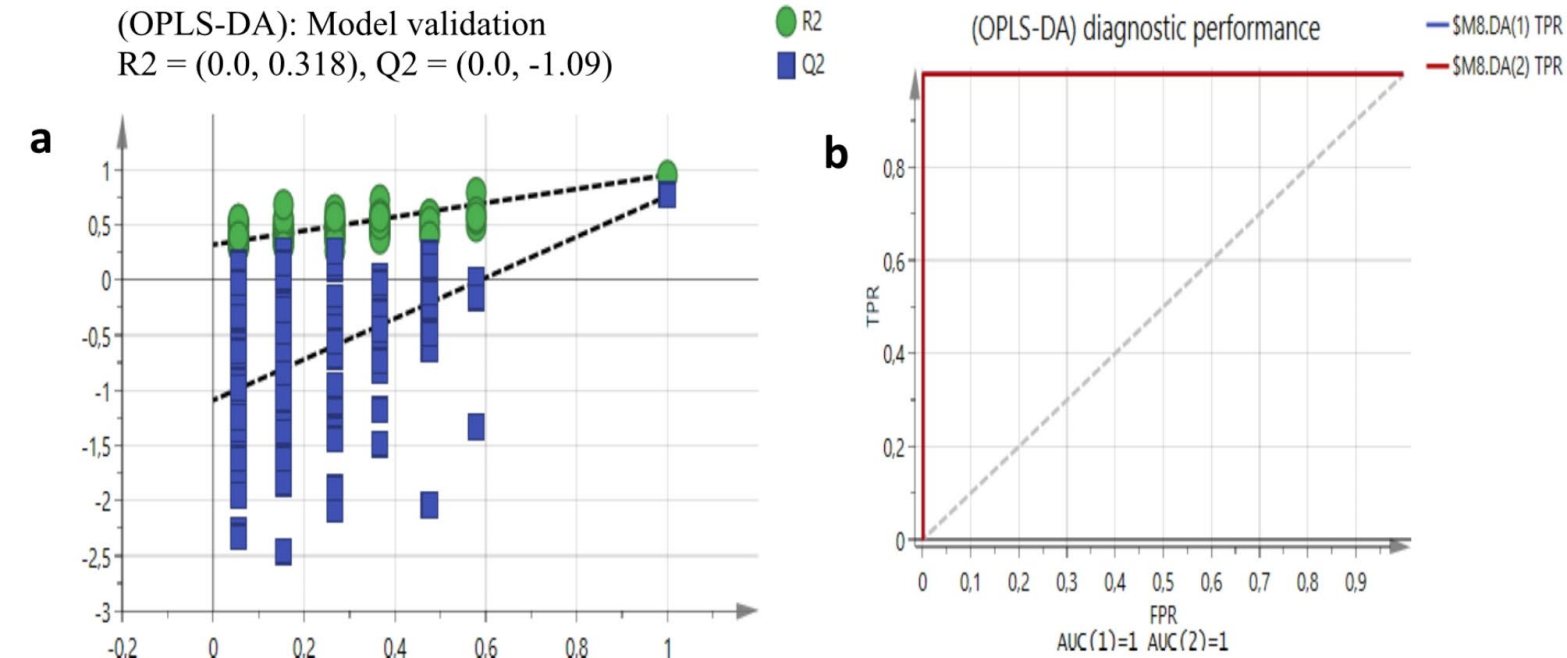
Statistical validation **a** OPLS-DA model using 120 permutation testing and diagnostic performance using ROC (AUC = 0.999) **b** Q2 intercept of -1.09 is less than 0.05 indicating a valid model

The constructed OPLS-DA discriminant model was further validated with the statistical interference analysis. To interactively analyse the predictor variable Y dependent on the known measured variable X and obtain statistics on the variables, one hundred and twenty permutation tests were conducted (Fig. 4a). The model’s dependability and degree of overfitting were established by examining the intercept of the fitting line formed by the computed values R2X and Q2, which correspond to every sample on the Y coordinate axis. According to Westerhuis et al. (2008), the model’s predictive ability increases with a larger Q2 value while the explanatory power increases with a higher value of R2X. The established OPLS-DA discriminant model was not over-fitted and had a strong predictive ability, despite the clear difference in predictability, as indicated by the negative intercept of the Q2 regression line. The cross-validated predictive residual (CV-ANOVA, p-value < 0.05) and Receiver-Operated Characteristic (ROC), which computes the area under the curve (AUC) was constructed (Fig. 4b).

**Fig. 5:**
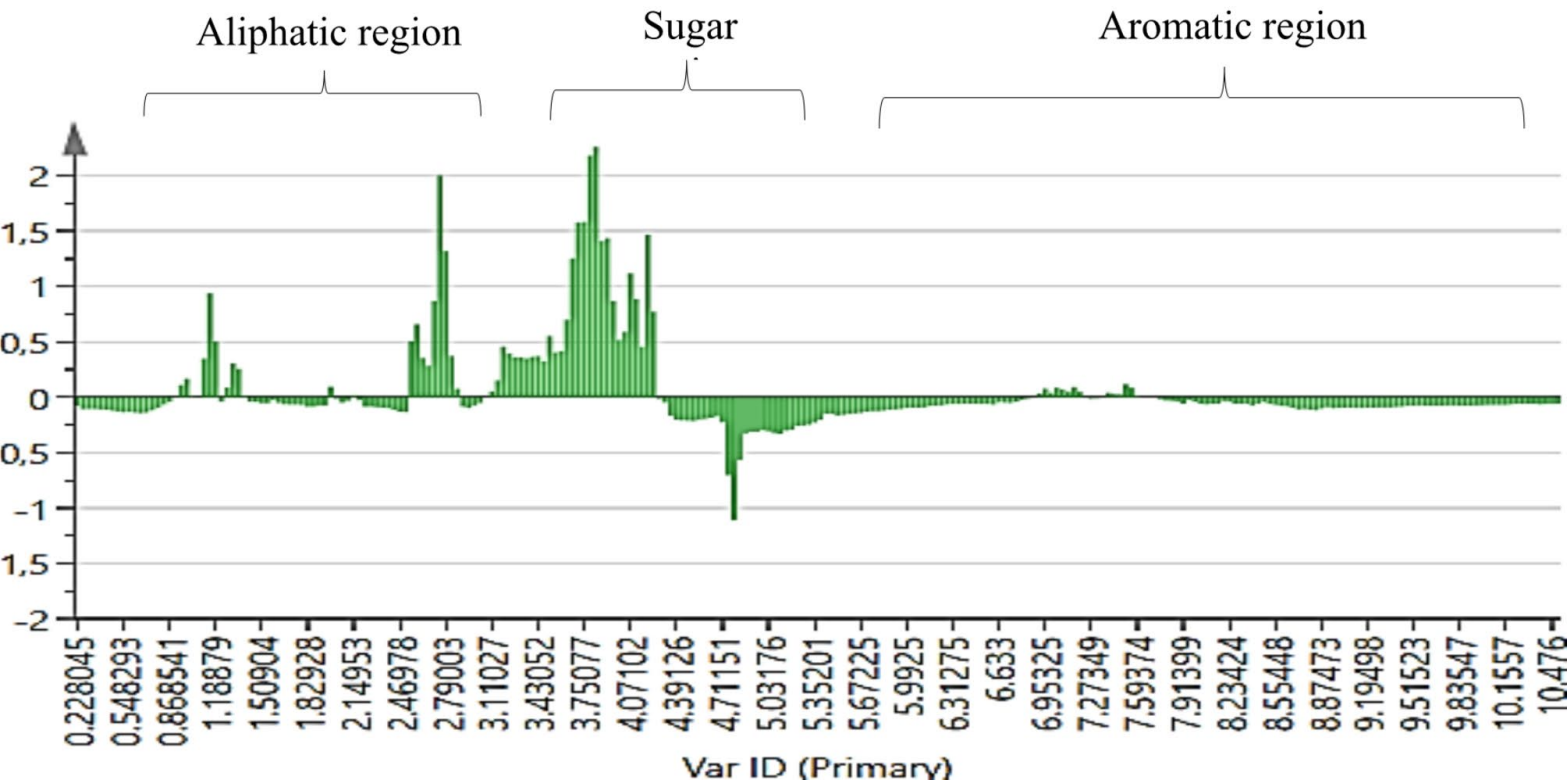
A contribution plot with significant 1 H NMR spectral regions responsible for the separation of *A. lettyae* from *A. longibracteata*. Positive scores are regions that are positively associated with the separation of *A. longibracteata* samples from the *A. lettyae* samples

Adopted from the supervised OPLS-DA results, the contribution plot (Fig. 5) revealed regions that are positively associated with the clustering of the *A. lettyae* and *A. longibracteata* samples. The sugar region in the peaks in the positive bars (*A. longibracteata)* of the contribution plot is more abundant followed by the aliphatic region with little intensity in the aromatic region. However, with *A. lettyae* (negative bars of contribution plot), the sugar and aromatic regions are more abundant than the aliphatic region. From the multivariate data analysis using SIMCA (version 14.0, Sartorius Stedim Data Analytics AB, Umetric, Umeå, Sweden), a visible discrimination factor between the two *Aloe* plants lies between the aliphatic and the aromatic regions, while the sugar region is the major source of the similarities in metabolites, although most chemical shifts remain unknown. Using Chenomx, the metabolites from *A. lettyae* and *A. longibracteata* were annotated in Table 1.

**Table 1:** Chenomx assisted annotations of differentially produced metabolites of *A. Lettyae* and *A*. *longibracteata*

Plant species	Metabolites	Chemical shift (ppm)	Concentration, (mM)
*Aloe lettyae*	4-Hydroxybenzoate	7.8 (d), 6.9(d)	0.019
	Adenine	8.2(s)	0.020
	Cadaverine	3.0(t), 1.7(m), 1.5(m)	0.053
	Putrescine	3.2(t), 1.8(m)	0.030
	Allantoin	8.0(s),7.3(s),6.0(s),5.4(s)	1.021
	Myo-inositol	3.6 (t), 4.1(t), 3.5(dd)	0.533
	Indole-3-acetate	7.6 (d), 7.5(d), 7.2(m), 3.6(d)	0.131
	Cis-Aconitate	5.7 (t), 3.1 (d)	0.034
	Erythritol	3.8(dd), 3.7 (m)	0.764
	Tyrosine	7.16(d),6.87(d),3.92(dd)	0.124
*Aloe longibracteata*	Aspartate	3.9(dd), 2.8 (dd), 2.7 (dd)	0.911
	Betaine	3.9 (s)	0.083
	Biotin	6.5 (s), 6.4(d)	0.908
	Chlorogenate	7.6(s), 7.2(d), 6.9(d), 4.3(q), 3.9 (q)	0.518
	Citrate	2.7(d), 2.5 (d)	0.377
	Tyrosine	7.16(d),6.87(d),3.92(dd)	0.387
	5-Hydroxy indole-3-acetate	7.4(d), 7.2(s),7.0(d),6.8 (dd),	0.076
	Arabinitol	3.9(m), 3.8(dd), 3.6(dd)	0.984
	Myo-inositol	3.6 (t), 4.1(t), 3.5(dd)	0.475
	Allantoin	8.0(s),7.3(s),6.0(s),5.4(s)	0.023
	Glycerate	4.0(dd), 3.8(dd), 3.7 (dd)	2.145
	β-Alanine	3.2(t), 2.6(t)	0.090

Peak multiplicity (s = singlet; d = doublet; t = triplet; dd = doublet of doublet; q = quartet; m = multiplet).

The Variable Important in Projection (VIP) scores (Supplementary Fig. S1) are used to effectively identify the key metabolites that are most responsible for chemical diversity and uniqueness between two *Aloe*. The VIP scores indicate the significance of each metabolite in distinguishing between the two *Aloe* based on their chemical composition. Higher VIP scores signify a greater influence of a specific metabolite in differentiating the two species. 3-Hydroxykynurenine has the highest VIP score (Supplementary Fig. S1) indicating the abundance of the metabolite varies significantly between the two species and is a major discriminating factor in their metabolite compositions. Compounds such as *N*-phenylacetyl, salicylurate, xanthurenate, and NADPH also have relatively high VIP scores, suggesting their levels or structural features contribute substantially to differentiating the chemical profiles of the two species. Metabolites with lower VIP scores such as tyrosine, *N*-acetylglutamine, and indole acetic acid (IAA) are less influential in distinguishing between the plant species based on their metabolite data. 

### Endophytic bacterial community distribution in the*Aloe*species

**Fig. 6:**
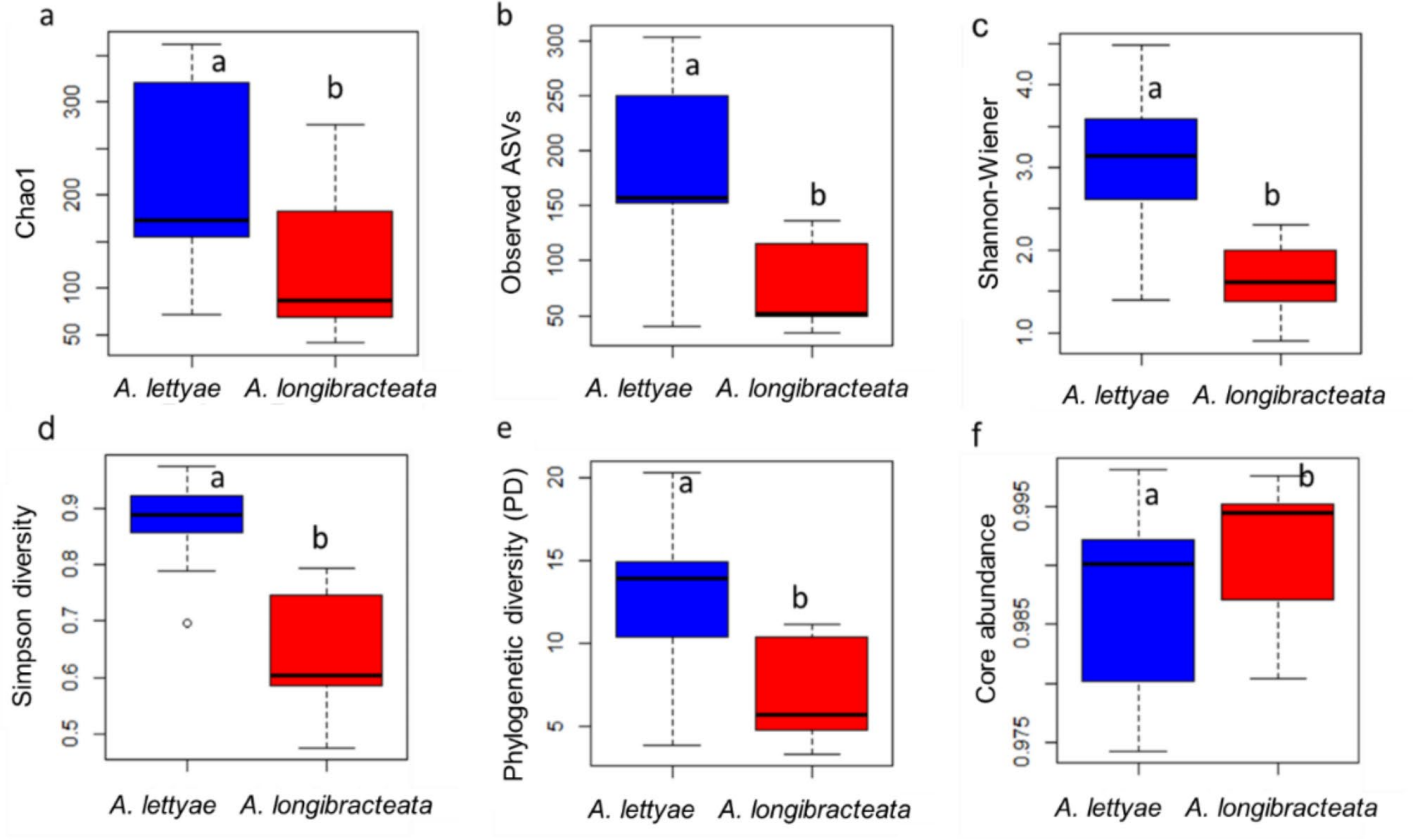
Alpha diversity measures across *A. lettyae* and *A. longibracteata*. **a** Chao1 **b** Observed ASVs **c** Shannon-Wiener diversity **d** Simpson diversity **e** Phylogenetic diversity (PD) **f** Core abundance

Shannon, Simpson, Observed ASVs, Chao1, phylogenetic diversity, and core abundance were significantly different (Mann-Whitney U test, *P* < 0.05) across the aloe species (Fig. 6). The *A. lettyae* species had a higher relative abundance and richness of bacterial endophytic communities compared to the *A. longibracteata* (Fig. 6a–e). The higher phylogenetically diverse index in *A. lettyae* indicates higher richness and diversity compared to the *A. longibracteata.* However, the core species were higher in the *A. longibracteata* compared to *A. lettyae*, suggesting a higher relative proportion of the core species in the former plant (Fig. 6f).

### Core and shared bacterial communities between the aloe species

The *A. lettyae* and *A. longibracteata* species differ in their number of unique and shared bacterial communities (Supplementary Fig. S2). The highest number of unique ASVs (40%) was observed in *A. lettyae* compared to *A. longibracteata* 28%, while both plant species shared 31% of the total bacterial communities. The shared and unique ASVs are taxonomically diverse with no dominant taxa identified between the aloe species.

### Endophytic bacterial community structure between the aloe species

**Fig. 7:**
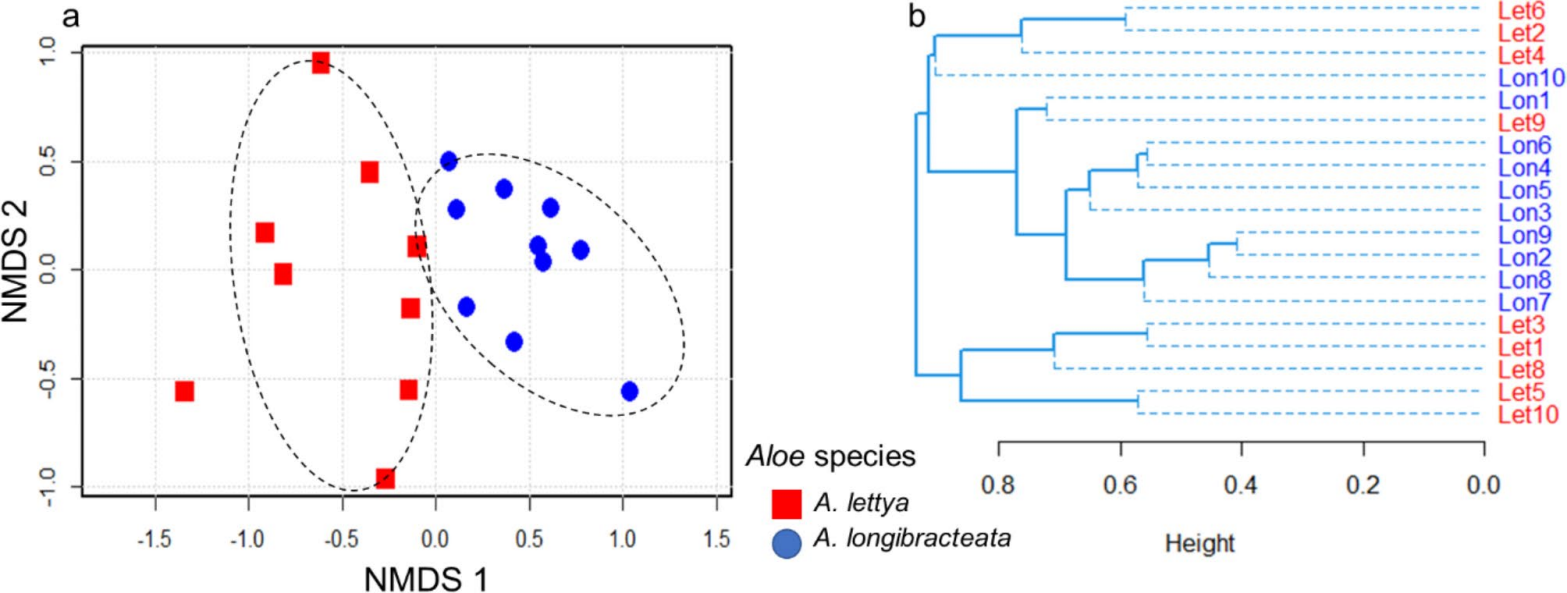
Bray-Curtis’s distance dissimilarity between endophytic bacterial communities of aloe species **a** Nonmetric multidimensional scaling plots and **b** cluster dendrogram. The dotted lines show the distances between species communities. The stress plot run was at 0.1941 (Supplementary Fig. S5)

The NMDS ordination analysis reveals that the endophytic bacterial communities of the aloe plants are well differentiated between the species (Fig. 7a). The bacterial communities of *A. lettyae* cluster separately from that of *A. longibracteata*. A similar trend was observed for the dendrogram plot (Fig. 7b). The PERMANOVA analysis suggests that the plant species had significant effects on the bacterial community composition (PERMANOVA; *P* < 0.05), and the dispersion test (PERMDISP, *P* > 0.05, Supplementary Table S1) was not significant, which infer the assumption of homogeneity was met. The non-significant PERMDISP indicates the dispersion between the plant species types may not have affected the observed variations in the bacterial communities of the species.

### Relative abundance of endophytic bacteria across the aloe species

**Fig. 8:**
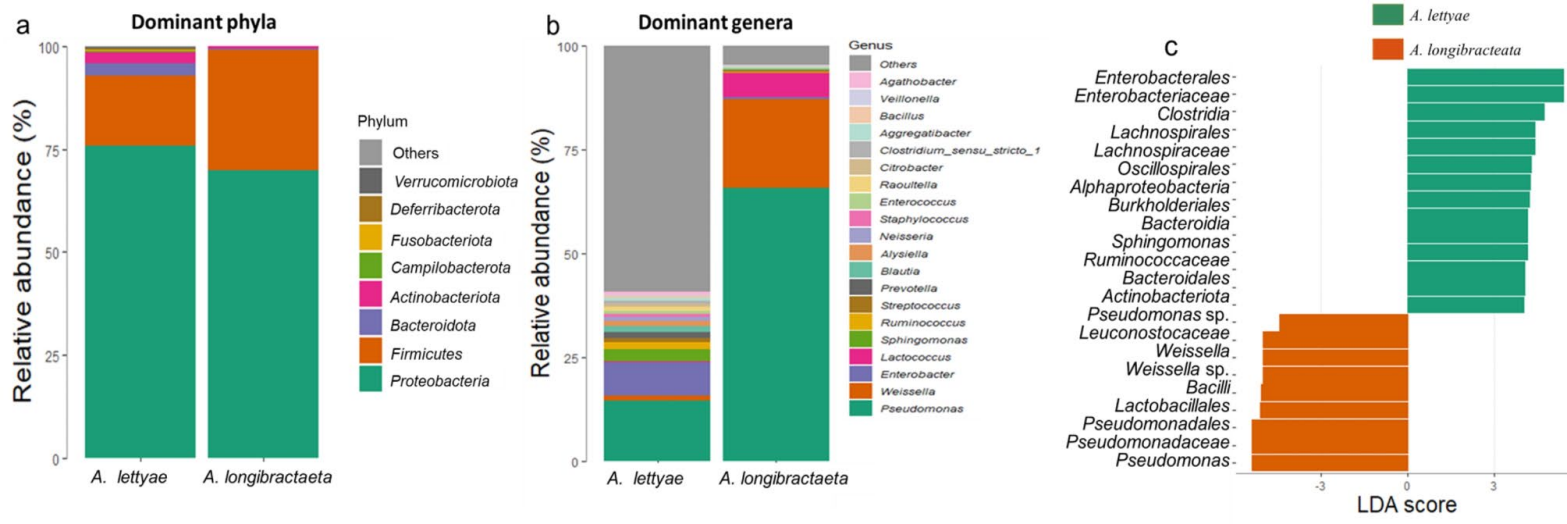
Relative abundance of endophytic bacterial community (> 1%) in *Aloe* spp. **a** Dominant phyla **b** Dominant genera **c** Bar plot from LEfSe analysis comparing the discriminant features between aloe species. The taxa level Phylum p_ to genus g_ is shown

The filtered reads were clustered into 1930 ASVs and the sequencing depth was confirmed adequate using the rarefaction curve (Supplementary Fig. S6). The dominant ASVs as shown by the taxonomic classification belong majorly to three phyla and seven genera (Fig. 8a and b), with *Proteobacteria* and *Firmicutes* being the most relatively abundant across the aloe species, representing over 80% of the total endophytic bacterial communities at the phyla level. *Actinobacteria* and *Bacteroidota* were more dominant in *A. lettyae. Pseudomonas*, *Weisella*, *Enterobacter*, *Lactococcus*,* Sphingomonas*, *Ruminococcus*,* Streptococcus* and *Prevotella* genera were the most relatively abundant bacterial communities across both species (Fig. 8b and c). However, their relative abundance differs with *A. lettyae* having the highest bacterial community richness. *A. longibracteata* had more abundance of *Pseudomonas*, *Weisella*, and *Lactococcus* compared to *A. lettyae*.

LEfSe analyses showed taxa differences from phylum to species with the absolute LDA score value > 2.76. *Enterobacterales*,* Clostridia*,* Lachnospirale* and *Oscillospirales* were discriminant in *A. lettyae*, while *Pseudomonas*, *Lactobacillales*,* Bacilli* and *Weisella* were discriminant in *A. longibracteata* (Fig. 8c).

### Correlation between key metabolites and endophytic bacterial communities of aloe species

**Fig. 9:**
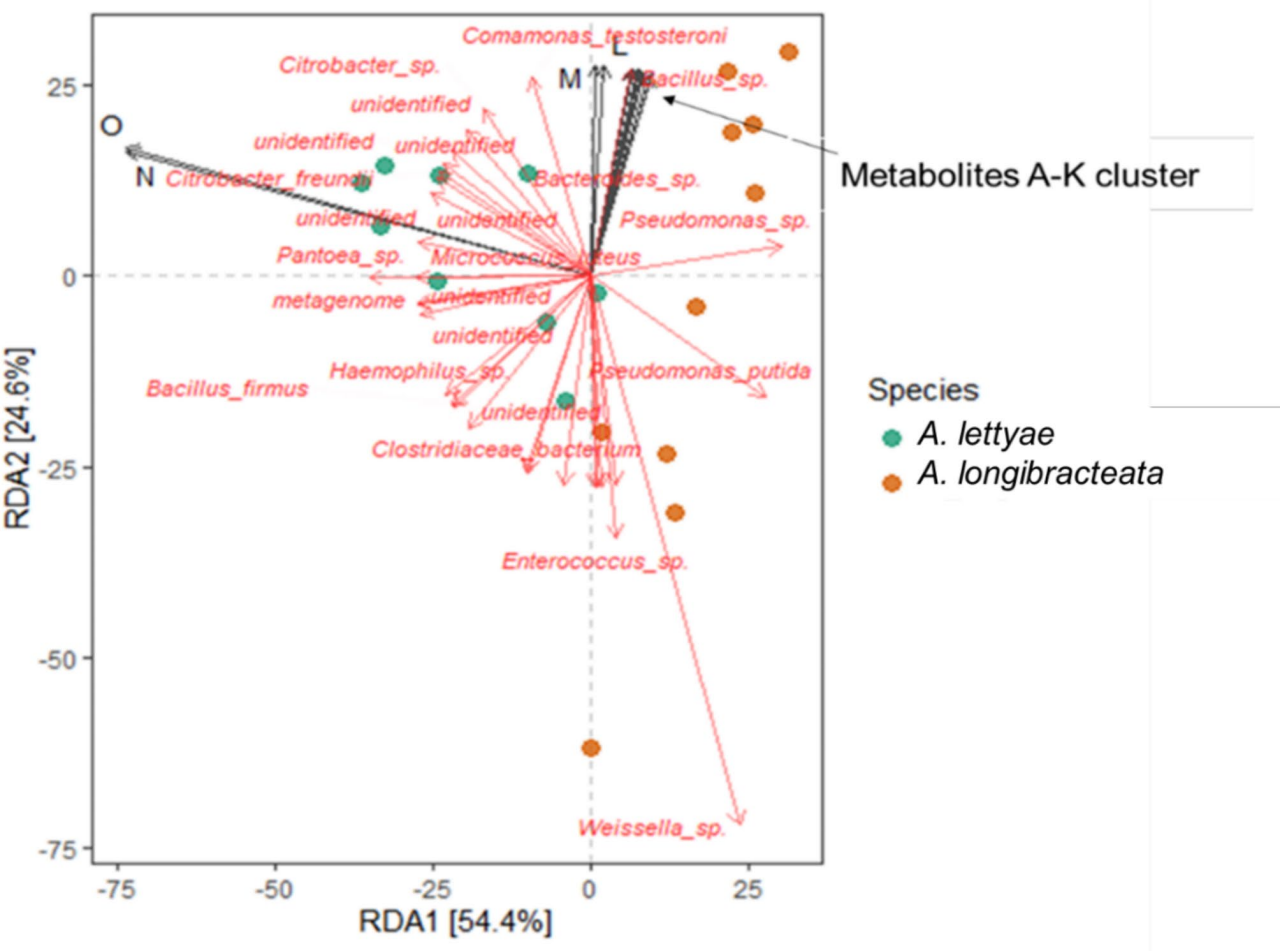
Redundancy analysis showing the correlation between endophytic bacterial communities and key metabolites of aloe plants. A: *N*-Acetylglutamine, B: 3-Hydroxyphenylacetate, C: Tyrosine, D: 3-Hydroxymandelate, E: NADPH, F: Salicylurate, G: 3-Hydroxykynurenine. H: N-Phenylacetylphenylalanine. I: *N*-Acetyltyrosine. J: 4-Hydroxyphenylacetate. K: Agmatine. L: 5-Hydroxyindole-3-acetate. M: Xanthurenate. N: Indole-3-Acetate. O: *N*-Acetylserotonin

The correlation analysis showed a clear separation between *A. lettyae* and *A. longibracteata*, with some of the metabolites exhibiting strong positive correlations with the microbial taxa (Fig. 9). The redundancy analysis (RDA) signifies that the plant metabolites contributed a negligible part of the variations (3%, R-squared adjusted value) observed in the bacterial community structure. Importantly, indole-3-acetate (N) and *N*-acetylserotonin (O) greatly impact the bacterial communities of *A. lettyae* and are largely associated with *Citrobacter*,* Micrococcus* and *Pantoea*. The L; 5-Hydroxyindole-3-acetate, and M; xanthurenate drive the endophytic bacterial communities in *A. longibracteata*, with high correlation with *Comamonas* and *Bacteroides* while the other metabolites (A-K cluster) were highly associated with *Bacillus*, *Comamonas* and *Bacteroides.* Metabolites of *A. lettyae* cluster more with many of the endophytic communities compared to the *A. longibracteata* and metabolites such as xanthurenate (M), *N*-acetylserotonin (O) and indole-3-acetate (N) have key human health importance.

Furthermore, the correlation analysis showed that 14 ASVs had a positive correlation with at least one of the differential metabolites while 36 ASVs were negatively correlated. Of note are ASV851 (*Staphylococcus*), ASV161 and ASV1503 (*Pseudomonas*), ASV340 (*Enterobacteriaceae*), ASV569 (*Weissella*) and ASV 731 (*Enterobacteriaceae*), which had a strong positive correlation with IAA and *N*-acetylserotonin (Supplementary Fig. S4). These ASVs, in addition to ASV1128 (Uncultured species), ASV1230 (*Weissella*), and ASV446 (*Pseudomonas*) were positively correlated with the other differential metabolites. The ASV1230 and ASV446, had a significant (*P* < 0.05) correlation with all the differential metabolites except for indole-3-acetic acid and *N*-acetylserotonin while ASV731, ASV340 and ASV997 in the family (*Enterobacteriaceae*), and ASV161 (*Pseudomonas*), had a significant correlation with all the metabolites, Similarly, ASV1128 had a significant (*P* < 0.05) correlation with all metabolites except 5-hydroxyindole-3-acetate, xanthurenate, indole-3-acetate and *N*-acetylserotonin (Supplementary Fig. S4).

### Metabolic profiling of endophytic bacterial communities in *Aloe* species

The PICRUSt2 predicted metabolic profiling of the endophytic bacteria in the aloe plants revealed 99.9% of the ASVs mapped with the KEGG with less than 0.6% of the gene sequences having above the maximum NSTI cut-off of 2. The total metabolic pathways and enzyme classification (EC) metabolic functions inferred were 395 and 2,064, respectively. A total of 6560 KEGG orthology (KO) was also predicted from all the ASVs. Predicted metabolic functions above 5% frequency at subclass 1 are cofactor, carrier, and vitamin biosynthesis (15.44%), amino acid biosynthesis (9.11%), aromatic compound degradation (8.86%), nucleoside and nucleotide biosynthesis (7.59%) carbohydrate biosynthesis (5.32%) and carbohydrate degradation (5.06%) (Supplementary Table S2). *A. lettyae* had the highest relative abundance of carbohydrate degradation, fatty-acid and lipid degradation, fermentation and glycolysis compared to *A. longibracteata* (Supplementary Fig. S3). The rest metabolic functions were higher for *A. longibracteata.*

**Fig. 10:**
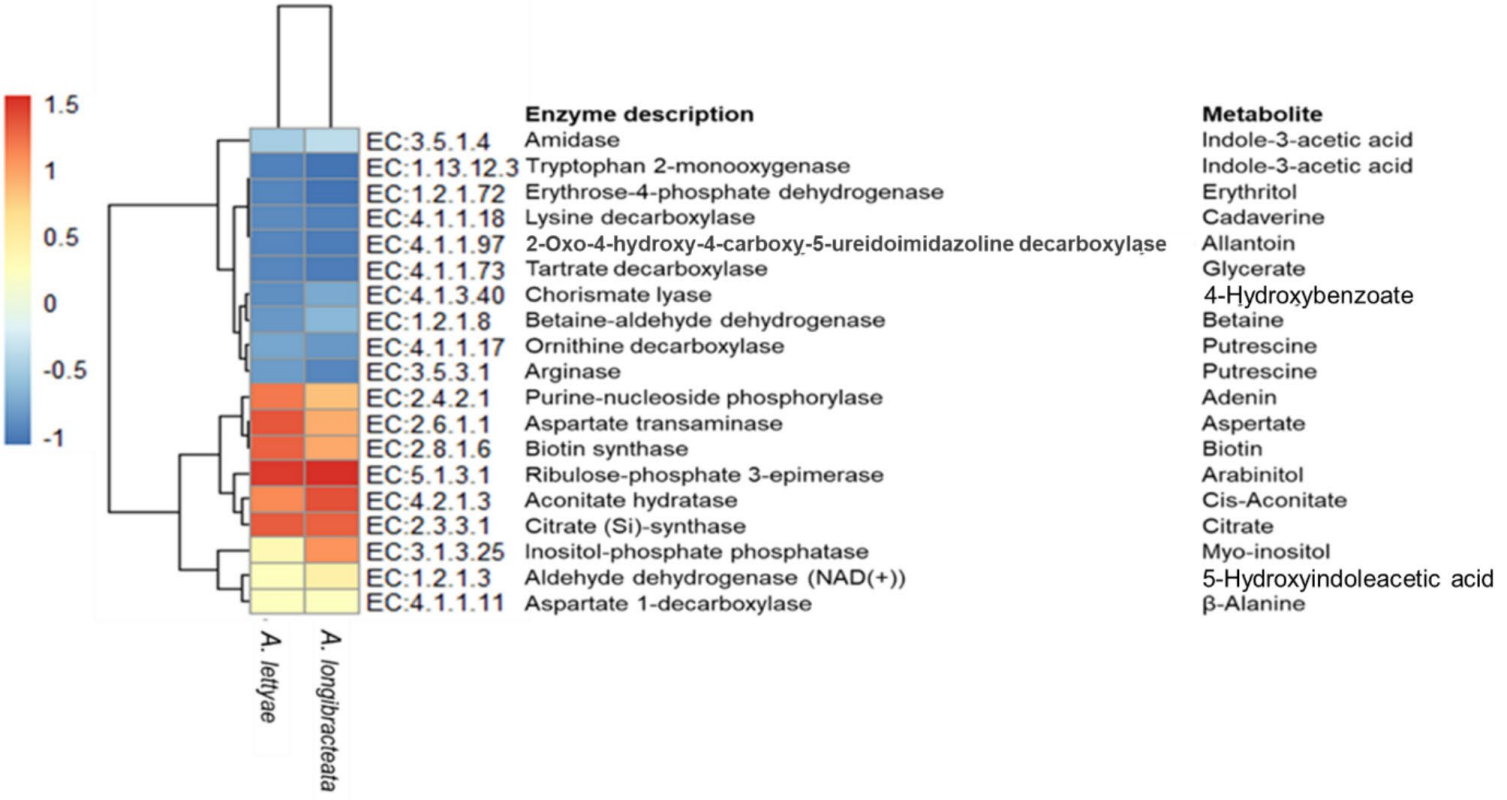
Variation of bacterial metabolic functional profile between *A. lettyea* and *A. longibracteata* showing enzymes involved in the pathways of some important metabolites of the endophytic bacteria in the aloe species

The endophytic bacterial communities of the aloes have the potential to produce metabolites analogous to those of the host plants. This is evident in their diverse enzymes contributing to different pathways in the degradation and biosynthesis of important host plant metabolites (Fig. 10). Enzymes involved in the biosynthesis of metabolites such as adenine, aspartate and biotin were more predicted in *A. lettyae* compared to *A. longibracteata*, which had a higher relative abundance of enzymes for cis-aconitate, myo-inositol, betaine, 5-hydroxyindoleacetic acid and 4-hydroxybenzoate (Fig. 10).

The presence of these enzymes in some of the endophytic bacteria may contribute to the host plant metabolite production. Similar to what was obtained for the plant metabolites, the enzyme participating in putrescine biosynthesis was highly predicted among endophytic bacteria of *A. lettyae*. Some of the endophytic bacteria found in this study, including *Pseudomonas*,* Bacillus*,* Lactococcus*,* Streptococcus* and *Enterobacter* possess various enzymes involved in the synthesis of some of the metabolites reported in the host plant. *Pseudomonas* and *Bacillus* are predicted to contribute to cadaverine and cis-aconitate production through the enzyme lysine decarboxylase and aconitate hydrolase, respectively. In addition, the genus *Streptococcus* could synthesise the enzyme ribulose-phosphate 3-epimerase in the pathway of arabinitol production.

## Discussion

Bioprocessing of medicinal plants has been a major strategy for sourcing metabolites used in drug synthesis and biotechnological applications (Sahoo et al. 2017). However, as some of the important plants are becoming extinct and classified as endangered species, it is important to find sustainable alternative sources of their metabolites and gain insight into their genetic components, including endophytic microbial communities before they are lost (Raimi and Adeleke 2021). Thus, this study evaluates the differential metabolites of endangered and unendangered aloe species and further correlates the results with the potential metabolic profile of the plant’s endophytic bacterial communities. The NMR spectroscopy was used to evaluate the variations in the metabolite composition of aloe species. NMR has been a major method for efficient analyses of plant extracts (Bilia 2006). Unlike HPLC, HPTLC, and capillary GC with specific detectors such as coupled systems (mass spectrophotometer), NMR is less time-consuming for simple and rapid chromatographic separation and can detect unknown metabolites that contribute to biological activity. (Bilia 2006). In addition, the high-throughput sequencing employed in this study provided an effective way to analyse all microbes present in a sample, allowing for easy annotation and classification of diverse microbes to obtain a complete microbial community composition (Kulski 2016).

### Aloe plants comprise diverse secondary metabolites of economic importance

Aloe plants are widely used for various purposes due to the beneficial properties of their secondary metabolites (Bajpai 2018). The metabolite annotation in this study revealed important phytohormones (IAA and 5-hydroxyindole-3-acetate) and phytochemicals (4-hydroxybenzoate, adenine, biotin, and citrate) from the aloe species. Previous studies have established the biological activities of tyrosine, allantoin, putrescine, IAA and alanine, which were also annotated in this study (Ahluwalia et al. 2021; Chelu et al. 2023; Kaur et al. 2021; Martínez-Sánchez et al. 2020). The results also showed that the aloes contain some analogous metabolites (Table 1), although in different concentrations, highlighting the feasibility of using *A. longibracteata* to replace *A. lettyae* due to its greater availability and lower conservation concerns. In this context, the study’s hypothesis that the use of *A. longibracteata* should be promoted over the endangered *A. lettyae* is validated, indicating an interesting avenue for sustainable resource management. The interchangeable use of *A. lettyae* and *A. longibracteata* is plausible due to their common metabolites, including allantoin, tyrosine, and myo-inositol. Extensively studied in other aloe species, these metabolites are associated with various uses in cosmeceuticals and human health management. Allantoin, known for its moisturizing properties, is commonly used in skin care products and pharmaceutical formulations (Talakoub et al. 2009). Tyrosine, acting as a precursor for neurotransmitters and thyroid hormones, plays crucial roles in mood regulation, cognitive function, and metabolism (Wang et al. 2012). Myo-inositol has been researched for its potential therapeutic effects on conditions such as depression, anxiety, and metabolic disorders (Vadnal et al. 1997). Due to the similarities in the morphological structures of most aloe plants, collectors may accidentally collect one species instead of the other. However, due to the shared biochemical chemistry of most aloe species, the intended uses are still realized. When considering the possibility of this substitution, it is crucial to consider factors beyond the presence of metabolites, such as bioactivity, quantity, and market demand to comprehensively assess the suitability of the aloes for industrial applications and conservation initiatives.

### The endophytic bacterial community of aloe plants differ between *Aloe* species

Although *A. lettyae* and *A. longibracteata* exhibit common metabolites, they also showed some distinct differences in their metabolic profiles due to various factors, reflecting the complexity of their metabolic pathways and the influence of environmental and genetic determinants. In our study, a clear distinction was observed in the endophytic bacterial community between *A. lettyae* and *A. longibracteata* (Fig. 7), which can significantly impact metabolite profiles (Chevrette et al. 2022; Woźniak et al. 2022). Different bacterial strains possess unique enzymatic capabilities and metabolic interactions with their host plants, influencing the synthesis, modification, or degradation of specific metabolites (Narayanan and Glick 2022). As a result, the variations in microbial composition could have contributed to the differences in the metabolite profiles of the aloes. Additionally, environmental factors such as soil composition, climate, and habitat conditions can exert selective pressures that shape metabolic adaptations in each species over time, influencing the production of specialized metabolites (Sampaio et al. 2016). Although not established in this study, the relationship between microbial and environmental factors underscores the complexity of metabolite production in aloe species, emphasizing the need for more comprehensive studies to elucidate the underlying mechanism driving metabolic diversity under different ecological factors.

### Secondary metabolites of aloes and their potential in human and crop health management

The aloes produced key metabolites with human health importance, such as 4-hydroxybenzoate and chlorogenate (Table 1) produced by *A. lettyae* and *A. longibracteata*, respectively. In a study by Kosová et al. (2015), 4-hydroxybenzoate was demonstrated to have comparable inhibitory activity to commercially used parabens against various pathogens, such as *Staphylococcus aureus* ATCC 6538, *Escherichia coli* ATCC 700,728, *Saccharomyces cerevisiae* DMF 1017 and *Fusarium culmorum* DMF 0103. This metabolite may also exhibit neuroprotective effects against Alzheimer’s disease (Winter et al. 2017). These dual properties underpin the significance of 4-hydroxybenzoate as an antimicrobial agent and as a candidate for neuroprotection against Alzheimer’s disease. Adenine, another metabolite from *A. lettyae* with human health benefits, is a key component of nucleic acids (DNA and RNA) and adenosine triphosphate (ATP), playing a critical role in cellular energy metabolism and genetic processes (Matsumoto et al. 1980). The deficiency of adenine contributes to severe combined immunodeficiency disorder (SCID) (Flinn and Gennery 2018). Chlorogenate, biotin and citrate are differentially abundant metabolites in *A. longibracteata*. Previous studies have revealed the antiviral effect of chlorogenic acid, a chlorogenate type metabolite against influenza A viruses on MDCK cells: A/Puerto Rico/8/1934(H1N1) and A/Beijing/32/92(H3N2) viruses with EC_50_ values of 44.87 µM and 62.33 µM, respectively (Ding et al. 2017). Biotin is an essential vitamin involved in various physiological processes such as energy metabolism, gene regulation, and histone modifications (Zempleni et al. 2008). Its deficiency can lead to metabolic disorders, skin rashes, and neurological symptoms (Mock 2017). The energy metabolism, bone health, and acid-base balance of citrate, as outlined by Costello et al. (2012), suggest that it is an anticoagulant and chelating agent for the treatment of certain diseases, which further underscores its potential therapeutic applications. Collectively, the findings reveal that *A. lettyae* and *A. longibracteata* species possess a rich reservoir of secondary metabolites with promising applications for human health and disease management.

Some of the annotated metabolites, including indole-3-acetate and its derivative 5-hydroxyindole-3-acetate are phytohormones, which play key roles in plant growth promotion (Raimi et al. 2017). These phytohormones enhance cell elongation, apical dominance, tissue differentiation, root initiation, and plants' ability to acquire water and nutrients from the soil (Fu et al. 2015). This growth-promotion property suggests that the metabolites of unendangered aloe species can be harnessed to enhance the yield and biomass of endangered aloe species. Interestingly, microbial endophytes also produce these plant hormones (Raimi and Adeleke 2023), emphasizing the need for further research into the endophytic microbial communities of aloe species and their abilities to synthesize host metabolites. Thus, aloe species and their microbial endophytes are useful in the conservation strategy of endangered aloe species and in increasing metabolite synthesis through plant biomass production and plant health management.

### Differential metabolites and bacterial community composition between *A. lettyae* and *A. longibracteata*

The NMR-based metabolomics showed that there are 15 differential metabolites between the two aloe species (Supplementary Fig. S1). Thirteen out of 15 metabolites from *A. longibracteata* had a significantly higher concentration compared to that of *A. lettyae*. On the other hand, the microbial community analysis indicated that *A. lettya*e harbours a more phylogenetically diverse bacterial community compared to *A. longibracteata*, suggesting higher diversity may not necessarily translate to more differential metabolites. It may be necessary to further study the *A. lettyae* and its complete microbiome before it disappears from the face of the earth, depriving us of the opportunity to leverage its unique bacterial community for beneficial purposes. The correlation analysis (Fig. 9) showed a high association between the metabolites and endophytic bacteria, providing additional evidence that the microbes could play a pivotal role as primary drivers of secondary metabolite production in the host plant (Prakash et al. 2019; Singh et al. 2019). Apine and Jadhav (2011) demonstrated that *Pantoea agglomerans*, a bacterium associated with plants, produced IAA. Similarly, the present study showed that *Pantoea* and *Citrobacter* exhibited a notable correlation with *N*-acetylserotonin and IAA, suggesting that the endophytes can influence the production of unique metabolites in the aloe plant.

Although there is currently no prior documented study on the bacterial community of *A. lettyae* and *A. longibracteata* species, the endophytic bacterial communities of *Aloe vera*, a commonly known *Aloe* species, have been extensively investigated, revealing a diverse spectrum of bacteria (da Silva et al. 2022; Swati et al. 2022). Akinsanya et al. (2015) and Silva et al. (2020) identified *Proteobacteria*,* Firmicutes*,* Bacteroides* and *Actinobacteria* as the dominant phyla, with specific genera such as *Pseudomonas*,* Bacillus* and *Enterobacter* being predominant. These bacteria have been reported to produce siderophores and antimicrobial compounds, indicating their potential in plant growth promotion and biocontrol of pathogens (Jha et al. 2011; Nutaratat et al. 2017; Saranraj et al. 2022). These endophytic bacteria can be employed to improve the growth, yield and productivity of aloe plants (Raimi and Adeleke 2023), thus increasing the availability of aloe biomass for biotechnological applications. There were high numbers of unclassified taxa (data not shown), which may suggest that the aloe endophytic bacterial communities are still underexplored with several unculturable species, presenting a source of potential novel isolates.

### Endophytic bacterial communities of aloe plants contribute to diverse metabolic pathways

The significance of correlating differential metabolites with dominant endophytic bacteria lies in unravelling the complex interplay between plant metabolism and microbial communities within the host. *Pseudomonas aeruginosa* has been found to produce agmatine, a metabolite of arginine, through the aguBA operon (Williams et al. 2010). This pathway is said to be induced during the stationary phase of growth and biofilm formation (Williams et al. 2010). The agmatine deiminase pathway, involved in the metabolism of agmatine and microbial putrescine biosynthesis, is characteristic of *Pseudomonas* species (Stalon and Mercenier 1984), a dominant genus reported in the aloe species in this study. Moreover, the use of C4-dicarboxylic acids, such as malate, by Bacteroides in N-fixation is regulated by amino acid excretion and polyhydroxybutyrate biosynthesis, with glutamate being an overflow product (Poole and Allaway 2000). *Comamonas testosteroni* has been found to produce tyrosine, a key amino acid involved in various biological processes (Brooks and Benisek 1994). This ability is possibly due to the presence of a distinct tyrosinase (TyrA), which is involved in melanin production in the related genus *Aeromonas* (Wan et al. 2009). The unique ability of endophytes to produce similar metabolites as the host suggests their potential as viable resources in microbial technology for the synthesis of various bioactive compounds. However, further study is necessary to substantiate the possibility of utilizing endophytes for metabolite production rather than relying solely on plant tissues, thus, offering a sustainable approach to utilizing plant resources (Mishra et al. 2021).

Very few studies have explored the biosynthetic pathways of secondary metabolites in aloe species to date (Kim et al. 2021; Ushasree et al. 2024). However, research on the endophytic communities of aloe plants has revealed a range of bacterial species such as B*acillus*,* Pseudomonas*, and *Enterobacter* being the dominant genera, contributing to various metabolic pathways (Akinsanya et al. 2015) and suggesting their application in microbial technology for the production of unique metabolites. *Bacteroides fragilis* and other species from this genus have been shown to metabolize tryptophan via the kynurenine pathway, resulting in the synthesis of xanthurenate alongside other tryptophan-derived metabolites (Agus et al. 2018; Bear 2023). These findings are consistent with the outcomes of our study, where xanthurenate was reported among the key metabolites of the aloe species.

Overall, this study presents the first comprehensive investigation into the metabolite profiles and endophytic bacterial communities of *A. lettyae* (endangered) and *A. longibracteata*. The identification of key metabolites, including phytohormones with plant growth-promoting potential and bioactive compounds with health benefits, underscores the value of the aloe species in sustainable agriculture and human health management. Given the endangered status of *A. lettyae*, the findings emphasize the urgency of preserving this unique species and its associated microbial communities. Additionally, the identification of shared metabolites and endophytic bacterial communities between the aloe species could facilitate the use of *A. longibracteata* instead of *A. lettyae*, thereby leading to the conservation and sustainable utilization of the unique biochemical resources of the endangered species.

## Electronic supplementary material

Below is the link to the electronic supplementary material.

Supplementary Material 1

## Data Availability

The sequences from this study have been submitted to the sequence read archives (SRA) under the SRA accession number PRJNA1098075 (http://www.ncbi.nlm.nih.gov/bioproject/1098075) as part of a BioProject in the National Centre for Biotechnological Information (NCBI).
