# Supplementary material for: Metabolomic profiling and 16 S rRNA metabarcoding of endophytes of two Aloe species revealed diverse metabolites
Source: AMB Express. 2024 Nov 8;14:122. doi: 10.1186/s13568-024-01784-3 (PMC11549271; doi:10.1186/s13568-024-01784-3)
Supplement: Supplementary file 1 — Supplementary Material 1 [file 13568_2024_1784_MOESM1_ESM.pdf]

Supplementary information

Metabolomic profiling and 16S rRNA metabarcoding of endophytes of two *Aloe* species revealed diverse metabolites

[a] Cynthia Marokane-Radebe, [a] Adekunle Raimi, [a, b] Stephen Amoo, [a] Rasheed Adeleke*

[a] Unit of Environmental Sciences and Management, North-West University. Potchefstroom 2520, South Africa

[b] Agricultural Research Council - Vegetables, Industrial and Medicinal Plants, Roodeplaat-Pretoria, 0001. South Africa.

*Corresponding author

Email: rasheed.adeleke@nwu.ac.za, Phone no: +27 018 299 2495

AMB Express

Supplementary Tables

Supplementary Table S1: PERMANOVA and PERMDISP analysis between *Aloe* species

Factor	PERMANOVA			PERMDISP
	DF	R2 (%)	*P*	*P*
Species type	1	19.947	0.001	0.058
Residue	17	80.053		

Statistical value is based on $P < 0.05$, DF, degree of freedom.

Supplementary Table S2: Predicted metabolic functions of *A. lettyae* and *A. longibracteata*

Subclass 1	Frequency	Percentage
Cofactor, -Carrier, -and-Vitamin-Biosynthesis	61	15.44
Amino-Acid-Biosynthesis	36	9.11
Aromatic-Compound-Degradation	35	8.89
Nucleoside-and-Nucleotide-Biosynthesis	30	7.59
Carbohydrate-Biosynthesis	21	5.32
Carbohydrate-Degradation	20	5.06
Fatty-Acid-and-Lipid-Biosynthesis	17	4.30
Cell-Structure-Biosynthesis	16	4.05
Amino-Acid-Degradation	15	3.80
Carboxylate-Degradation	14	3.54
Fermentation	11	2.78
Secondary-Metabolite-Biosynthesis	11	2.78
Amine-and-Polyamine-Degradation	10	2.53
C1-Compound-Utilization-and-Assimilation	10	2.53
Super pathways	10	2.53
Inorganic-Nutrient-Metabolism	9	2.28
Nucleoside and Nucleotide Degradation	9	2.28
Secondary-Metabolite-Degradation	6	1.52
TCA-cycle	6	1.52
Tetrapyrrole-Biosynthesis	6	1.52
Amine-and-Polyamine-Biosynthesis	5	1.27
Generation-of-Precursor-Metabolites-and-Energy	5	1.27
Alcohol-Degradation	4	1.01
Fatty-Acid-and-Lipid-Degradation	4	1.01
Glycolysis	3	0.76
Other-Biosynthesis	3	0.76
Polyprenyl-Biosynthesis	3	0.76
Aromatic-Compound-Biosynthesis	2	0.51
Degradation/Utilization/Assimilation---Other	2	0.51
Nucleic-Acid-Processing	2	0.51
Pentose-Phosphate-Pathways	2	0.51
Aldehyde-Degradation	1	0.25
Aminoacyl-tRNA-Charging	1	0.25
Electron-Transfer-Chains	1	0.25
Energy-metabolism	1	0.25
Interconversion	1	0.25
Metabolic-Regulator-Biosynthesis	1	0.25
Respiration	1	0.25

Supplementary Figures

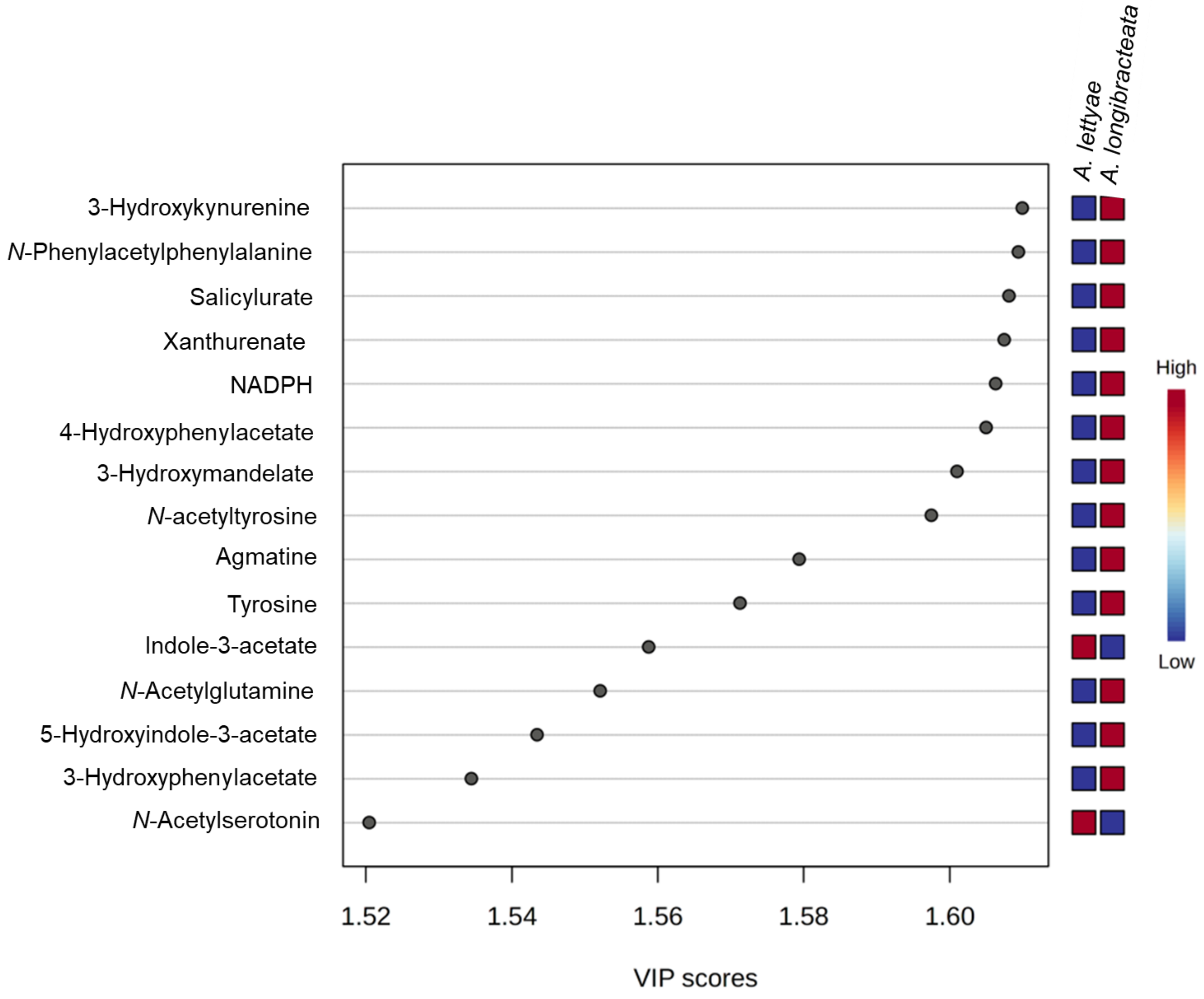

Supplementary Fig. S1. Variables Important in Projection (VIP) scores representing metabolites between the two *Aloe* species. The metabolites are listed from top to bottom as; 3-hydroxykynurenine, *N*-phenylacetylphenylalanine, salicylurate, xanthurenate, NADPH, 4-hydroxyphenylacetate, 3-hydroxymandelate, *N*-acetyltyrosine, agmatine, tyrosine, indole-3-acetate (IAA), *N*-acetylglutamine, 5-hydroxyindole-3-acetate, 3-hydroxyphenylacetate and *N*-acetylserotonin. The selected metabolites were those with VIP > 1. The heatmap on the right displays red and blue boxes, indicating, respectively high and low abundance ratios of the corresponding metabolites in *A. lettyae* and *A. longibracteata*

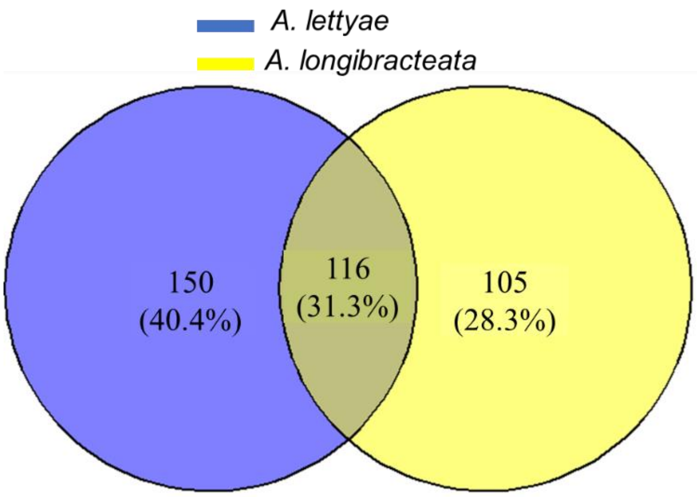

Supplementary Fig. S2. Shared and unique species in the bacterial communities between the *Aloe* species

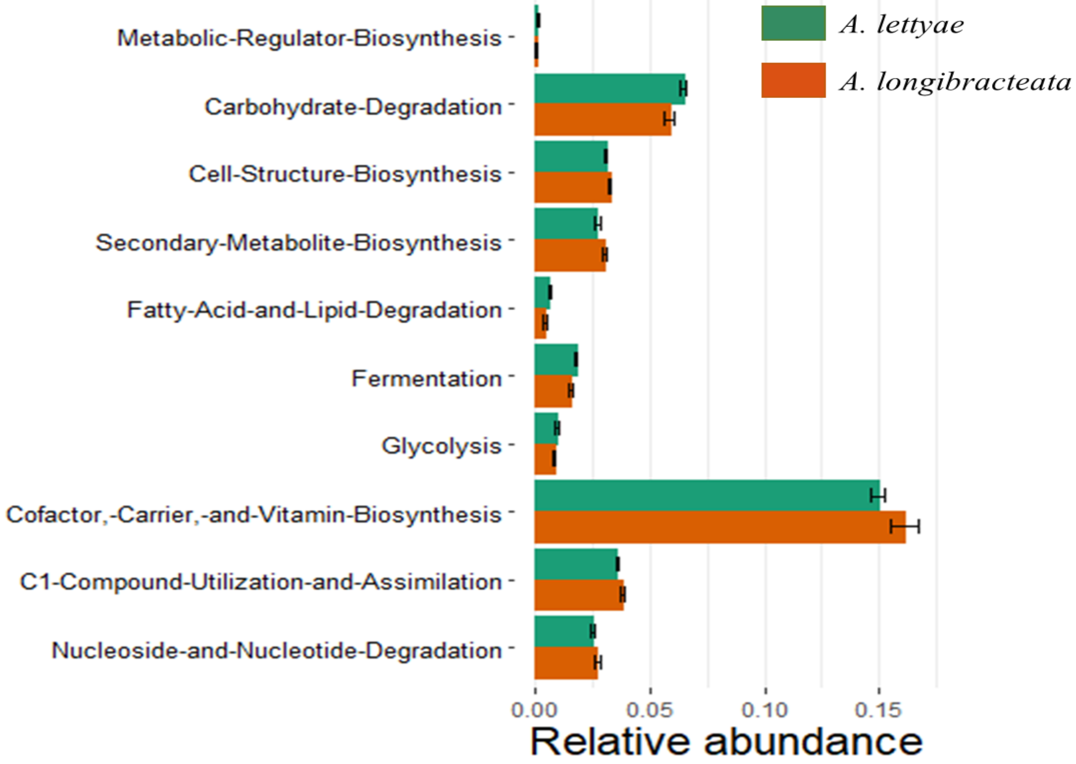

Supplementary Fig. S3. Relative abundance of the endophytic bacterial functions in the *Aloe* species

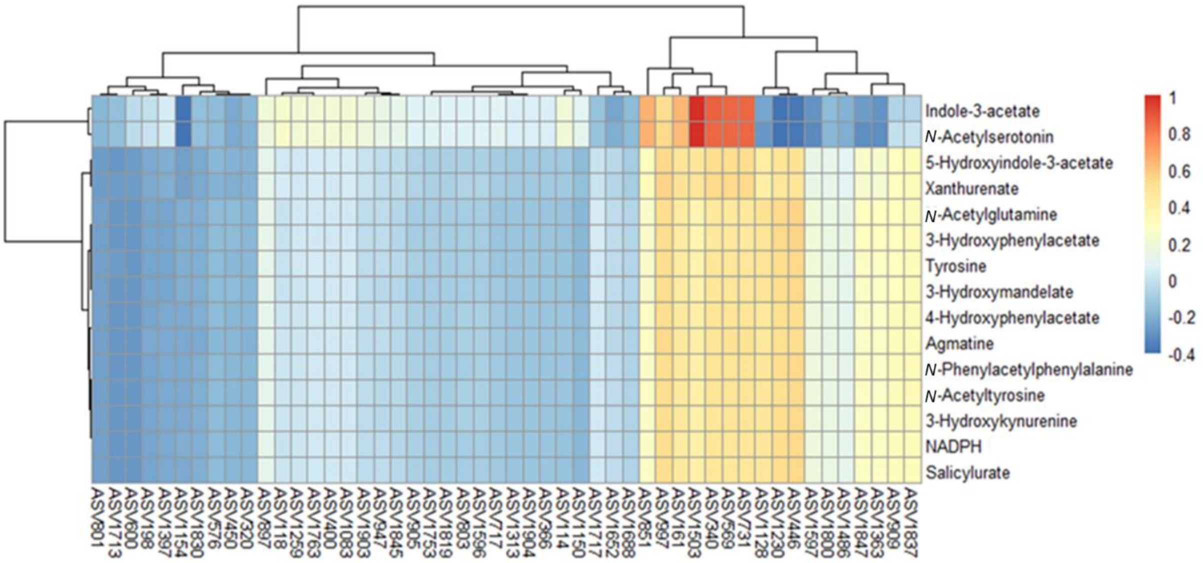

Supplementary Fig. S4. Correlation between differential metabolites and top 50 dominant endophytic bacteria of *Aloe* plants. The red and blue colours indicate positive and negative correlations, respectively. The colour depth indicates strong (deep colour) and weak (light colour) and correlation $P < 0.05$.

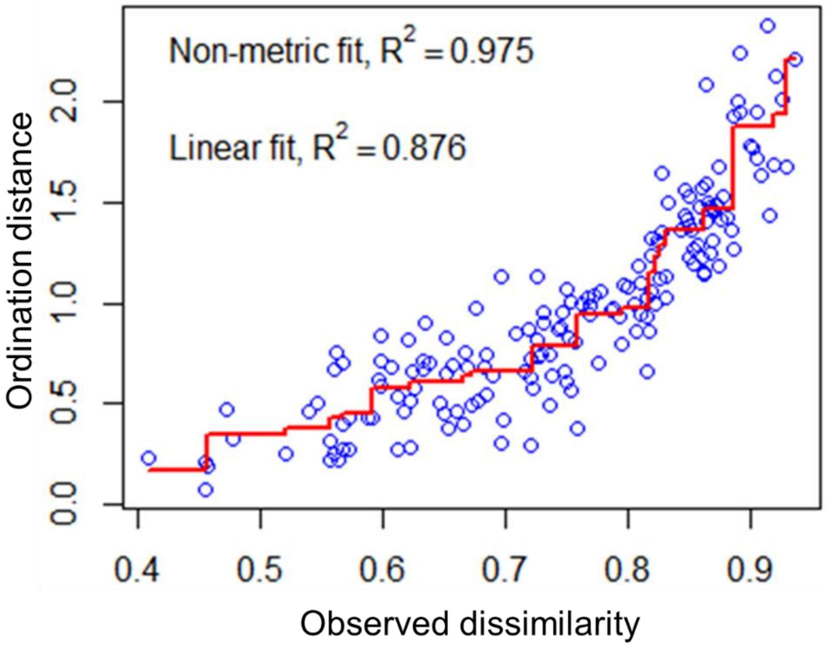

Supplementary Fig. S5. Stress plot for the nonmetric multidimensional scaling (NMDS) plot. The stress run was at 0.1941.

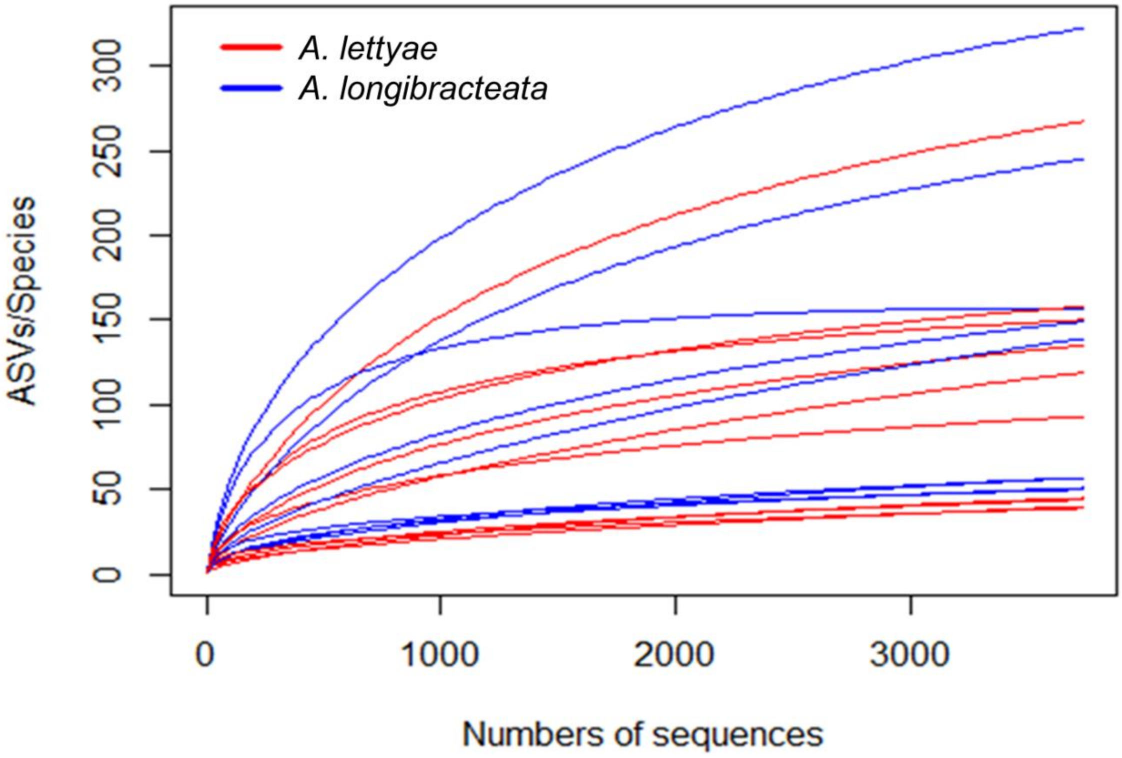

Supplementary Fig. S6. Rarefaction curve for sequencing reads from *Aloe* species.
